# microRNA input into a neural ultradian oscillator controls emergence and timing of alternative cell states

**DOI:** 10.1038/ncomms4399

**Published:** 2014-03-04

**Authors:** Marc Goodfellow, Nicholas E. Phillips, Cerys Manning, Tobias Galla, Nancy Papalopulu

**Affiliations:** 1 Faculty of Life Sciences, Michael Smith Building, The University of Manchester, Oxford Road, Manchester M13 9PT, UK; 2 Theoretical Physics, School of Physics and Astronomy, The University of Manchester, Manchester M13 9PL, UK; 3 Present address: College of Engineering, Mathematics and Physical Sciences, University of Exeter, Exeter, Devon EX4 4QF, UK

## Abstract

Progenitor maintenance, timed differentiation and the potential to enter quiescence are three fundamental processes that underlie the development of any organ system. In the nervous system, progenitor cells show short-period oscillations in the expression of the transcriptional repressor Hes1, while neurons and quiescent progenitors show stable low and high levels of Hes1, respectively. Here we use experimental data to develop a mathematical model of the double-negative interaction between *Hes1* and a microRNA, miR-9, with the aim of understanding how cells transition from one state to another. We show that the input of miR-9 into the Hes1 oscillator tunes its oscillatory dynamics, and endows the system with bistability and the ability to measure time to differentiation. Our results suggest that a relatively simple and widespread network of cross-repressive interactions provides a unifying framework for progenitor maintenance, the timing of differentiation and the emergence of alternative cell states.

## Abstract (web summary)

Hes1 is an important regulator of progenitor maintenance and timed differentiation, which shows oscillatory expression. Here, the authors combine experimental data and mathematical modelling to show that the interaction between miRNA-9 and Hes1 can predict progenitor transition from one cell state to another, as well as the timing of this transition.

## Graphical abstract

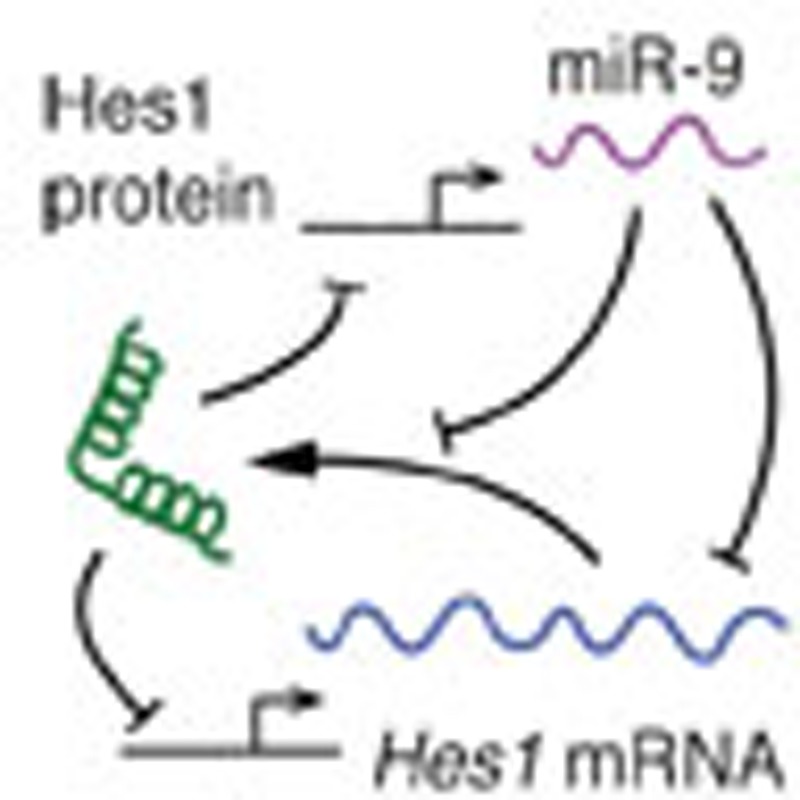

The nervous system is built by progenitor cells that divide either symmetrically or asymmetrically to give rise to neurons and glia^1^,^2^. Differentiated cells appear in a timed fashion, and although the timing of generation of different cell types is not as stereotypical as previously thought^3^, timing of differentiation is an important element in the correct development of the nervous system^4^. Some progenitors also need to replenish their population by symmetric proliferative or asymmetric divisions, whereby at least one of the daughter cells remains a progenitor. Such maintenance is imperative to avoid the premature depletion of the progenitor pool and to allow the nervous system to reach its final size. In addition, progenitors that remain at the end of neurogenesis may be transformed into quiescent progenitors that are able to generate neurons in the adult and be re-activated upon injury or neuronal loss^1^. Thus, the timing of differentiation, progenitor maintenance and the adoption of alternative cell states (that is, differentiation or quiescence) are three fundamental principles that underlie the development of the nervous system.

Experimental studies over several decades have elucidated some of the key molecular regulators of these processes. For example, Hes1, a basic helix-loop-helix transcriptional repressor activated in response to Notch signalling, has been shown to modulate the progenitor state. *Hes1* knockout mice show premature neuronal differentiation accompanied by early progenitor depletion, while *Hes1* overexpression prevents neuronal differentiation^5^,^6^. However, it is unclear how progenitor maintenance is co-ordinated with the timing of differentiation, the acquisition of alternative cells states and whether there is an underlying unifying mechanism for these processes.

The development of advanced live imaging techniques has allowed some hypotheses to be formulated in attempt to answer these questions. For example, the expression of *Hes1* has been shown to be much more dynamic than previously thought, displaying short-period (ultradian) oscillations in progenitor cells of different tissues^7^,^8^,^9^,^10^. Thus, based on a combination of experimental data and imaging of expression dynamics in normal development, it has been proposed that the Hes1 oscillatory state is necessary for the maintenance of progenitors, whereas sustained low or high levels are associated with differentiation or quiescence, respectively^6^. This hypothesis led us to suggest that understanding the mechanisms by which cells transition from the oscillatory state into sustained high or low levels of Hes1 may provide the long-sought unifying mechanism for co-ordination of the basic neurogenic processes.

Oscillations are predominantly caused by Hes1 autorepression, coupled with messenger RNA (mRNA) and protein instability^11^,^12^,^13^. Although mRNA instability is an essential component of oscillatory dynamics, the mechanisms of its regulation were unknown and therefore, mRNA degradation rate parameters were fixed in early models (with the exception of ref. 14). It was subsequently shown by us, and others, that miR-9 regulates the stability of *Hes1* mRNA and related genes in several model systems^9^,^15^,^16^,^17^,^18^ and is transcriptionally repressed by Hes1 in the mouse^9^ or Her6 in zebrafish^17^. We hypothesized that the slow degradation rate of mature miR-9 can increase the degradation of *Hes1* mRNA over time, leading to an exit from oscillations. Thus, we suggested that the double-negative feedback loop of miR-9 and Hes1 provides a mechanism for the exit of oscillations with an embedded self-limited timer^9^.

Here we use mathematical modelling to analyse the potential of this simple, but fundamental, transcription factor/microRNA (miRNA) network to explain the aforementioned principles of nervous system development. The mathematical model incorporates the effects of miRNAs on both mRNA stability and translational repression, using recent experimental results to constrain its parameters. We then demonstrate that Hes1/miR-9 interactions can lead to high or low Hes1 levels depending upon the strength of repressive interactions. Furthermore, these levels can be reached by transient oscillations, the lengths of which represent the amount of time that a cell spends in the progenitor state. Finally, we demonstrate that initial concentrations of miR-9 anticipates the future cell state choice, in terms of differentiation or quiescence, defined by low or high stable Hes1 levels. This is due to the emergence of bistability in the model, which is brought about by the introduction of miR-9. Thus, a relatively simple network of mRNA–miRNA interactions is capable of shaping the timing and fate choice of neural progenitors. The provision of a cell autonomous but tuneable timing mechanism reconciles the influence of intrinsic and extrinsic factors that govern the timing of differentiation. Furthermore, our model provides a unifying framework for progenitor maintenance, the timing of differentiation and the acquisition of a differentiated state or quiescence, thus accounting for some of the fundamental principles of neurogenesis.

## Results

### Computational model of the Hes1–miR-9 interaction network

**Figure 1:**
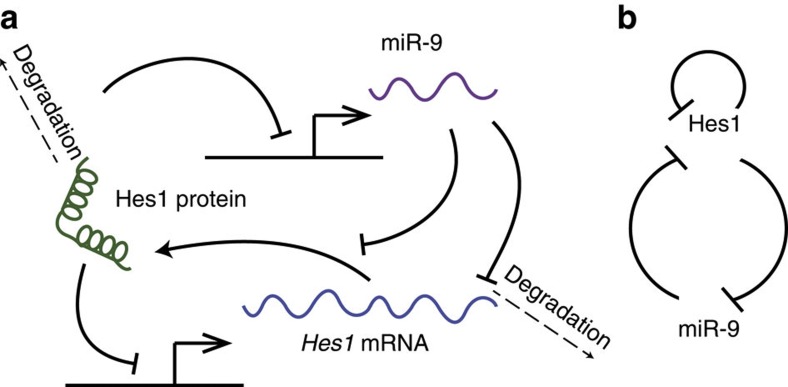
Experimentally determined network interactions between Hes1 and miR-9. (**a**) Detailed visualization of the Hes1/miR-9 network. Solid arrows indicate production, whereas flat line ends represent repressive interactions. (**b**) Simplified network motif.

In Fig. 1, we provide a visual representation of the network of interactions between Hes1 and miR-9, based on experimental data^9^. In addition to the autorepression of *Hes1* transcription by Hes1 protein, the incorporation of miR-9 leads to three new network interactions, namely the repression of miR-9 production by Hes1 protein, miR-9-mediated changes in mRNA degradation rate and putative miR-9 repression of Hes1 protein translation^9^,^18^,^19^,^20^. We use delay differential equations to track the change in relative amounts of Hes1 protein (*p*), mRNA (*m*) and miR-9 (*r*) over time arising due to ‘production’ and ‘degradation’ factors. The equations governing the dynamics of these three species are described sequentially below.

### The influence of miR-9 on *Hes1* mRNA production

Equation (1) describes the temporal change of *Hes1* mRNA concentration.


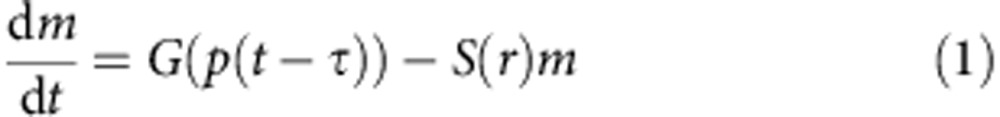


The autoinhibition of Hes1 acting as a repressor of transcription is implemented via the function *G*, the form of which is taken directly from previous models^12^,^13^,^14^ as follows:


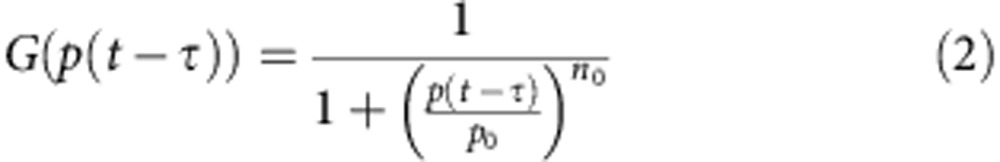


As levels of *p*(*t*−*τ*) increase (where *t*−*τ* accounts for the delay in protein production due to transcription and translation), autoinhibition increases because *G* decreases to small positive values and hence the total production term is reduced. The transition from minimal to maximal repression is governed by the shape of the function, *G*, which is determined by two parameters, *p*_0_ and *n*_0_. The parameter *p*_0_ represents the amount of protein required to reduce *Hes1* transcription by half and therefore embodies the relative strength of repression: low values of *p*_0_ imply that low values of protein can have a repressive effect, whereas high values for *p*_0_ imply large amounts of protein are required for transcription to be attenuated (Supplementary Fig. 1). The parameter *n*_0_ determines the steepness of *G* and has previously been described as a Hill coefficient indicating, for example, the degree of co-operativity in the repressive interaction^12^. Here, we describe it as a more abstract term that simply alters the shape of this function, with higher values of *n*_0_ making the transition from minimal to maximal repression more step-like, as shown in Supplementary Fig. 1b. In order to demonstrate the possible effects on the form of repression that arise from changes in *p*_0_ and *n*_0_, we provide examples in Supplementary Fig. 1.

The inclusion of miR-9 in the model means that the degradation term for *Hes1* mRNA (*m*) is now a dynamic function of miR-9 (*r*) levels. This function is informed by the experimental results of ref. 9, in which the authors measured the half-life of *Hes1* mRNA in wild-type cells as well as in cells injected with pre-miR-9 or containing *Hes1* 3′UTR with mutated binding sites, thus diminishing the effect of miR-9 (ref. 9). These results can be incorporated directly into our model since approximate bounds are provided for the half-life of *Hes1* mRNA when miR-9 is at high, low or intermediate levels. The results suggest that *Hes1* mRNA half-life is bounded between ~20 min for high levels of miR-9 and 35 min for low levels of miR-9, with the wild-type value in between these bounds. Taking this into account, we propose the following functional form for the role of miR-9 in altering *Hes1* mRNA half-life:


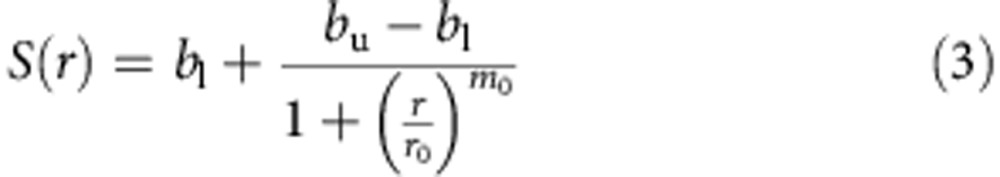


The general form of *S*(*r*) is chosen to be equivalent to that of *G* so that its shape is controlled by strength and shape parameters (here *r*_0_ and *m*_0_) (Supplementary Fig. 1). The parameters *b*_l_ and *b*_u_ impose lower and upper bounds for *Hes1* mRNA half-life and are fixed to ln(2)/20 min^−1^ and ln(2)/35 min^−1^, respectively.

### The influence of miR-9 on Hes1 protein production

Equation (4) describes the temporal change of Hes1 protein (*p*) levels.


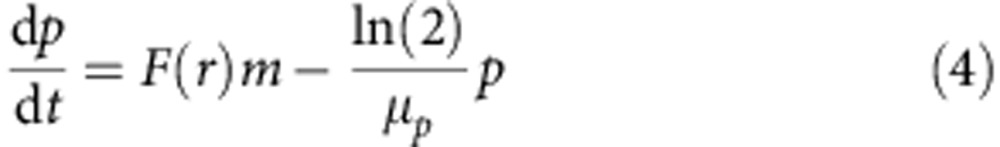


We have incorporated a new function, *F*(*r*), to capture the attenuation of protein production by miR-9. We retain the general form of *G* so that the strength and shape of translational repression (here governed by the parameters *r*_1_ and *m*_1_, respectively) can be tuned as follows:


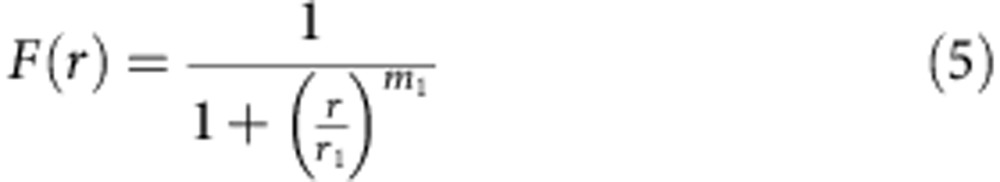


These equations allow us to study how the presence of miR-9 affects the amount of Hes1 protein by examining its effect on mRNA degradation independently from its effects on translation repression (by setting *F*(*r*)=1). Doing so renders Hes1 protein production independent of translational repression by miR-9.

Taken together, equations (1) and (4) represent a description of the Hes1 oscillator in which the effect of miR-9 levels on its dynamics can be explored by treating the levels of miRNA, *r*, as a parameter. By setting *r* to certain values and evaluating the dynamics of this reduced system (henceforth referred to as the ‘two-variable’ system; see below) one can generate a snapshot view of the way in which *r* can affect the Hes1 oscillator. However, this view of the system neglects the feedback from *p* onto *r*, hence a third equation tracking the dynamics of *r* was developed.

### The dynamics of miR-9 production

We introduce a new variable representing the levels of miR-9, *r*, and track its dynamics, taking into account the repression of miR-9 transcription by Hes1 as follows:


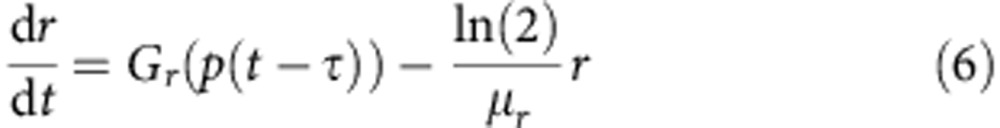


In equation (6), we assume that a constant upstream activation of miR-9 is attenuated due to the repressive activity of Hes1 protein. This repression is provided by the function *G*_*r*_, which follows the same general form as *G*. We allow for the case that repression by Hes1 acts differently at the miR-9 and *Hes1* loci by introducing different parameters for the strength and shape of repression in *G*_*r*_, that is, *p*_1_ and *n*_1_ as follows:


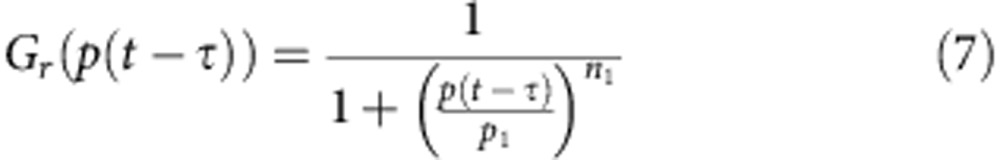


Note that *G*_*r*_ is shaped by *p*_1_ and *n*_1_ in the same way that *G* is shaped by *p*_0_ and *n*_0_. Setting *μ*_*r*_ (the half-life of miR-9) to high values replicates the high stability of miR-9 observed experimentally^9^.

Degradation terms in the model are expressed as half-lives by introducing the factor ln(2). The parameters *μ*_*p*_ and *μ*_*r*_ are therefore the half-lives of Hes1 protein and miR-9, respectively. The half-life of *Hes1* mRNA is affected by miR-9, and hence is given in the model by the function ln(2)/S(r).

In summary, the equations governing the system of Hes1 and miR-9 interactions are as follows:


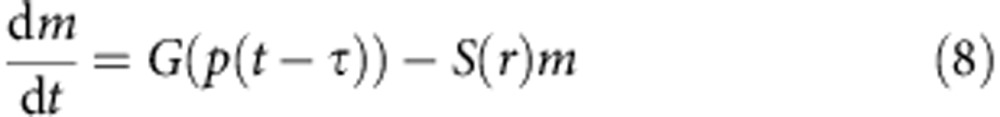



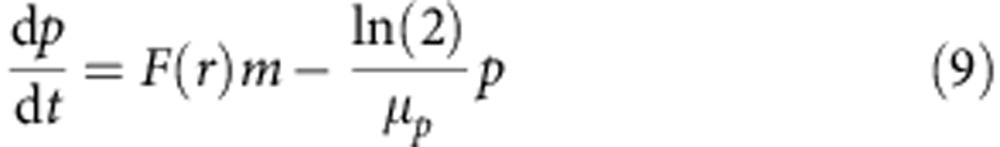



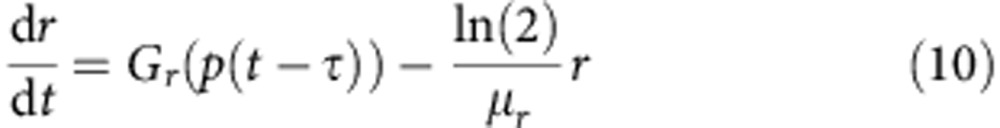


The system described by equations (8) and (9) is referred to as the two-variable model, while equations (8, 9, 10) are referred to as the full model.

### Model assumptions

A full list of parameters and their default values is given in Table 1, and a summary of the equations is given in Supplementary Note 1. The abstract formulation of the system we employ is based upon the following assumptions. We neglect the dynamics of external activating factors and focus instead on the internal dynamics of the Hes1/miR-9 system under the assumption that *Hes1* and miR-9 transcription are constitutively active when not repressed. Note that these activation factors are absorbed into the strength parameters of the repression functions^12^. To support our focus on intracellular mechanisms, we use bioluminescence imaging to demonstrate the persistence of oscillations in sparsely plated c17.2 neural progenitor cells in Supplementary Fig. 2.

The dynamics of free, mature miR-9 are lumped into a single variable, *r*, thus neglecting the pre- and mature versions of this species. We therefore do not account for differential stability of pre- and mature miR-9 or the time delay in the production of the active molecule. Degradation of free miR-9, together with association and dissociation of the miR-9/*Hes1* complex, are lumped into the degradation term for *r*. Stability of free miR-9 is kept high by assuming that the net effect of this degradation is small (that is, *μ*_*r*_ is assumed to take large values). Four inhibitory interactions are modelled by decreasing functions of substrate (*p* or *r*); (i) Hes1 protein inhibiting *Hes1* mRNA (*G*) (ii) Hes1 protein inhibiting miR-9 transcription (*G*_*r*_), (iii) miR-9 inhibiting *Hes1* mRNA transcription (*S*) and (iv) miR-9 inhibiting Hes1 translation (*F*). In the absence of specific information regarding the shape of these functions, we assume that they are governed by a function comprising parameters that control the shape (*n*_0_, *n*_1_, *m*_0_, *m*_1_) and strength (*p*_0_, *p*_1_, *r*_0_, *r*_1_) of inhibition.

### Model oscillations occur for experimental mRNA half-lives

Experimental results suggest that Hes1 protein oscillations should exist for half-lives of *Hes1* mRNA within the bounds of ~20 and 35 min. At values more extreme than these, oscillations are not sustained^9^,^18^. To discover whether our mathematical model recapitulates this experimental observation, we examined the presence of oscillations due to changes in the mRNA half-life, given by *S*(*r*). We focused initially on the case in which miR-9 acts only to reduce the stability of *Hes1* mRNA^9^, by setting *F*(*r*)=1 to negate the effects of *r* on translation of Hes1 protein (*p*) in the two-variable model (equations (8) and (9)).

**Figure 2:**
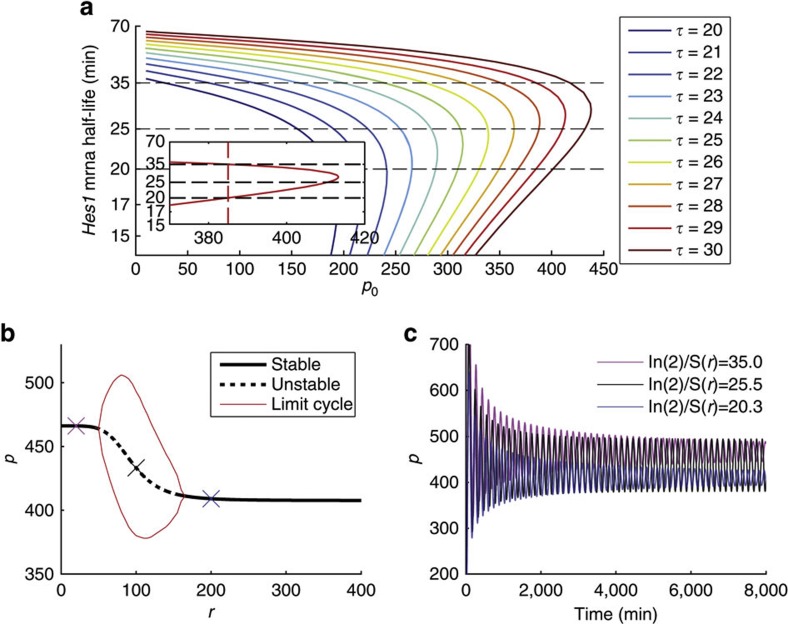
Presence of oscillations in the model when miR-9 acts only to affect the stability of *Hes1* mRNA. (**a**) Different combinations of *τ*, *p*_0_ and *S*(*r*) are used to test for the presence of oscillations. The different coloured lines denote Hopf bifurcations for different values of *τ* as indicated. A Hopf bifurcation denotes the transition of a system from a stable to an unstable, oscillatory state or *vice versa*, as a parameter of the system is varied. Fixed points exist to the right of the curves whereas oscillations are present to the left. The inset shows the curve for *τ*=29. The dashed red line indicates the value of *p*_0_ for which two Hopf bifurcations exist for mRNA half-lives of ~35 and 20 min. (**b**) A window of oscillations emerges for changes in *r*, with *p*_0_ fixed at 390 and *τ*=29 min. (**c**) Example time series when *r* is fixed to the values given by crosses in (**b**). These *r* values give rise to the half-lives (ln(2)/*S*(*r*)) as indicated. *n*_0_=5, *μ*_*p*_=22 min, *b*_l_=ln(2)/20 min^−1^, *b*_u_=ln(2)/35 min^−1^, *r*_0_=100, *m*_0_=5.

The parameter that defines the steepness of *Hes1* transcriptional autorepression, *n*_0_, was fixed to a value that has been shown to generate oscillations (that is, *n*_0_=5 (ref. 12), but the relative strength of transcriptional autorepression by Hes1 protein, *p*_0_, and the time delay in protein production, *τ*, were varied as shown in Fig. 2. The delay, *τ*, was allowed to take values in the physiologically plausible range of 20–30 min^21^. Figure 2 shows that, for each value of *p*_0_, oscillations exist as the half-life of *Hes1* mRNA (ln(2)/*S*(*r*)) is decreased. Furthermore, when *τ*=29 min, a window of oscillations arises that lies within the experimentally determined values of mRNA stability when *p*_0_ is around 390 (arbitrary units) (inset of Fig. 2a,b). Example time series are shown in Fig. 2c. In contrast to the sustained oscillations when *Hes1* mRNA half-life is around wild-type levels (ln(2)/*S*(*r*)=27 min (ref. 9)), oscillations dampen for parameters chosen outside the half-life boundaries reported in ref. 9.

**Figure 3:**
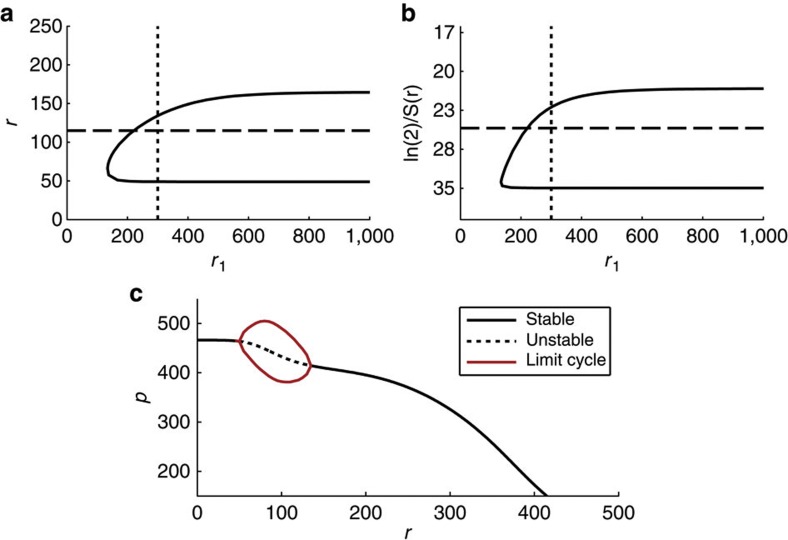
Presence of oscillations in the two-variable model when *r* acts via both mRNA degradation and translational repression. (**a**) Shows the location of Hopf bifurcations as *r*_1_ and *r* are varied. Here, oscillations exist to the right of the curve. The horizontal line indicates the value of *r* for which ln(2)/*S*(*r*)=25. The vertical dashed line indicates a value of *r*_1_=300. (**b**) as (**a**) but with *y* axis plotted in terms of mRNA half-life (ln(2)/*S*(*r*)). (**c**) A window of oscillations emerges for changes in *r*. The addition of translational repression allows a lower steady state for high *r*. Other parameters are *p*_0_=390, *τ*=29 min, *b*_l_=ln(2)/20 min^−1^, *b*_u_=ln(2)/35 min^−1^, *n*_0_=*m*_0_=*m*_1_=5, *r*_0_=100, *μ*_*p*_=22 min.

Although the effect of miR-9 on *Hes1* mRNA stability has been experimentally demonstrated, it is increasingly clear that miRNAs exert a combined effect on mRNA stability and translation^22^,^23^,^24^. To account for a combined action on *Hes1* mRNA degradation and translational repression, we introduced *F*(*r*) into the two-variable model. In this scenario, as *r* is varied over increasing positive values, the two repressive effects of *r*, that is, the degradation of *Hes1* mRNA and inhibition of the translation of Hes1 protein, act simultaneously. Supplementary Fig. 3 demonstrates that several parameter sets allow the generation of sustained oscillations under these circumstances. Furthermore, Fig. 3a,b demonstrate that an oscillatory window that contains the wild-type mRNA half-life value exists, in line with the results of Bonev *et al*.^9^, when the strength of the translational repression is relatively small (large *r*_1_) and the steepness of the repressive interactions relatively high (*m*_0_=*m*_1_=5). These results show that our model recapitulates the experimentally observed window of oscillations^9^ either by mRNA degradation acting alone or in combination with translational repression.

In summary, we have demonstrated that the experimentally observed window of oscillations can be present in the model for relevant values of the *Hes1* mRNA degradation rate. In the remainder of the paper, we investigate the effects of incorporating miR-9 interactions into the network. We fix *τ*, *p*_0_, *m*_0_, *m*_1_, *r*_0_ and *r*_1_ to the values given in Table 1, which represents a set of values for which the alignment with experiments holds. Other possible parameter values can be used, as demonstrated in Figs 2 and Supplementary Fig. 3 but these would not necessarily allow the model to match experimental observations.

### Attaining low values of Hes1

Experimental evidence shows that differentiated neurons are characterized by low stable levels of Hes1 protein^25^. We were therefore particularly interested in defining the conditions under which this dynamic network achieves low stable levels of Hes1. We analysed the two-variable model and focused initially on the case that miR-9 acts only to reduce the stability of *Hes1* mRNA by setting *F*(*r*) to 1, thus removing the influence of miRNA on the protein production rate. The stability and steady-state values of Hes1 protein were calculated and are shown in Fig. 2b. It can be seen that the system displays an oscillatory window (unstable dynamics, or ‘limit cycle’) while the final protein levels on either side of the oscillatory window are stable, either high or low. However, the accumulation of miR-9 can reduce the levels of Hes1 protein only by ~12% due to its action on mRNA degradation alone (Fig. 2b). A difference in concentration of this magnitude has been shown to be capable of mediating different cell states in the context of morphogens in *Drosophila* embryos^26^. However, neurons express very low or no levels of Hes1 (refs 25, 27); therefore, it is unclear whether such a small change would be sufficient in the developing nervous system. We therefore sought to determine the conditions that allow for a greater degree of attenuation of Hes1 protein, by allowing miR-9 to repress *Hes1* translation (that is, by allowing *F*(*r*) to vary). In this case, since *F*(*r*) can take values close to zero, the steady-state value of *p* can also, in principle, reach values close to zero. Thus, with translation repression acting alongside mRNA degradation, miR-9 is able to reduce Hes1 to lower stable state levels than when miR-9 acts solely to increase the degradation rate of *Hes1* mRNA (Fig. 3c, compare with Fig. 2b). The exact steady-state value of *p* attained depends upon all of the parameters of the system. We henceforth focus on the case that *p* is close to zero at high levels of *r*, though we note that a continuum of steady-state values of *p* can be attained by adjusting the parameters of the system.

### Including miR-9 in the Hes1 oscillator leads to bistability

Progenitors give rise to differentiated neurons but may also become quiescent, or slowly proliferating cells. Slowly proliferating, non-neurogenic progenitors, are found in boundary regions of the central nervous system and they are characterized by high stable levels of Hes1 (ref. 25). Progenitors may also enter a quiescent or slowly dividing state at the end of embryonic neurogenesis^1^,^28^. We therefore asked whether our model can lead to a mechanistic understanding of how this cell state is attained. Comparing the levels of *p* at either side of the oscillatory window in Fig. 3c demonstrates that higher steady-state levels of Hes1 protein are obtained when miR-9 levels are low (and hence *Hes1* mRNA half-life longer). However, this analysis does not take into account the mechanisms by which different miR-9 levels are attained. The findings of ref. 9 highlight that miR-9 is a dynamic component of the network and, therefore, we introduced *r* as a dynamic variable in the model. We assume that the production of miR-9 is constitutive but repressed by Hes1 (ref. 9) as described in equation (10) of the full Hes1/miR-9 network. The sum of the production and degradation rates determine whether miR-9 levels increase or decrease over time.

**Figure 4:**
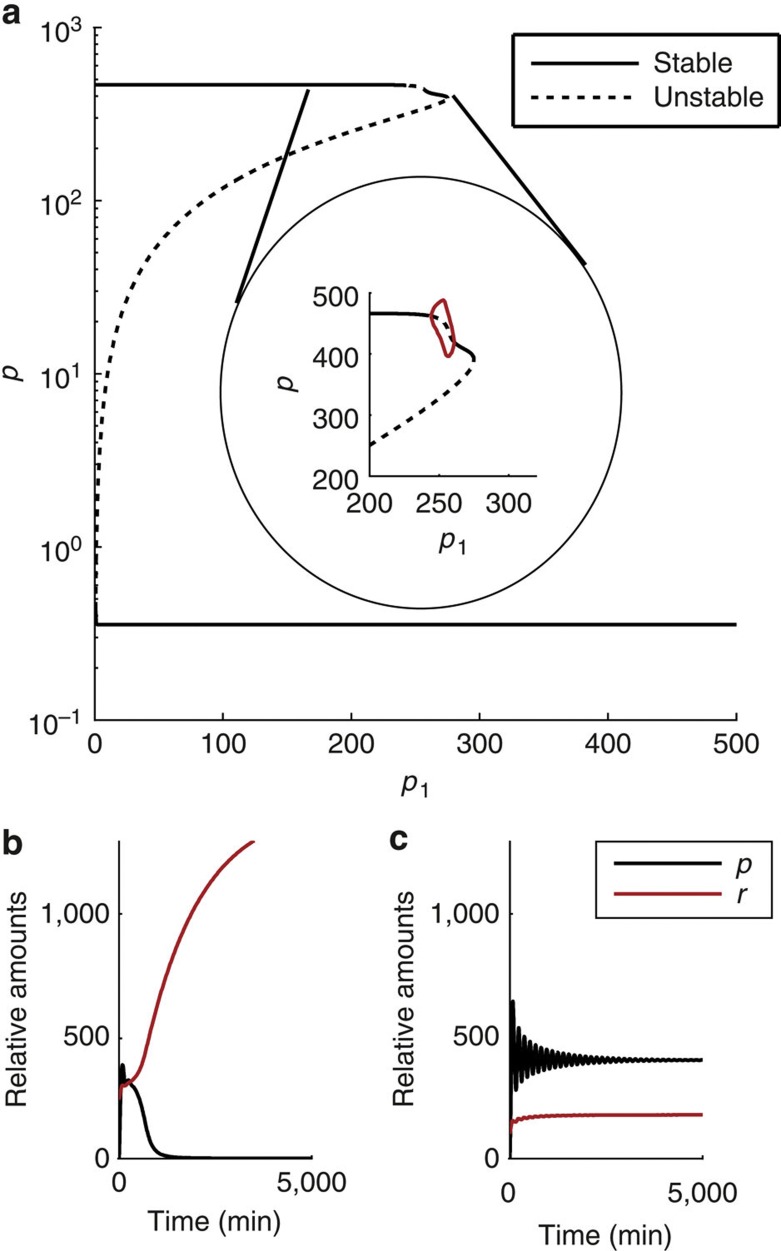
Oscillations and different steady-state levels of Hes1 in the full system for changes in the strength of repression of miR-9 by Hes1 (*p*_1_). (**a**) Shows a bifurcation diagram for changes in *p*_1_, with protein levels (*p*) given as output on the *y* axis. The circled area shows a magnified region of parameter space that contains oscillations (limit cycle maxima and minima given by red lines). (**b**,**c**) Show example of time series for a case of bistability (*p*_1_=272). The initial conditions (giving a constant history vector) are (**b**) *m*(0)=0, *p*(0)=0, *r*(0)=240 and (**c**) *m*(0)=0, *p*(0)=0, *r*(0)=100. Other parameters are *p*_0_=390, *τ*=29 min, *b*_l_=ln(2)/20 min^−1^, *b*_u_=ln(2)/35 min^−1^, *n*_0_=*n*_1_=*m*_0_=*m*_1_=5, *r*_0_=100, *r*_1_=300, *μ*_*p*_=22 min, *μ*_*r*_=1,000 min.

Figure 4a shows a bifurcation diagram for changes in the strength of repression of miR-9 by Hes1 (*p*_1_) in the full model. It can be seen that there are two branches of stable fixed points, which represent the high and low stable values of Hes1 described earlier. These states are produced in the model in an intuitive way due to changes in *p*_1_. When the strength of repression of miR-9 by Hes1 is low (high *p*_1_), miR-9 accumulates due to weak repression by Hes1 and this leads to a stable state with low Hes1 (*p*). However, as the strength of repression of miR-9 by Hes1 increases (decreasing *p*_1_), a new stable steady state emerges that has high Hes1 levels.

Figure 4a also shows that the network interactions of the full model give rise to a state of bistability between the high and low Hes1 states. Bistability (here due to fold bifurcations) means that, for identical parameter settings, the model can attain either sustained high or sustained low levels of Hes1 depending upon the levels of *m*, *p* and *r* that it is initiated with. Example time series for a bistable situation are shown in Fig. 4b,c. Figure 4b demonstrates that initially high levels of miR-9 (*r*) accumulate and maintain the suppression of Hes1 (*p*). In contrast, initially low levels of miR-9 (*r*) remain low, and lead to higher, stable levels of Hes1.

**Figure 5:**
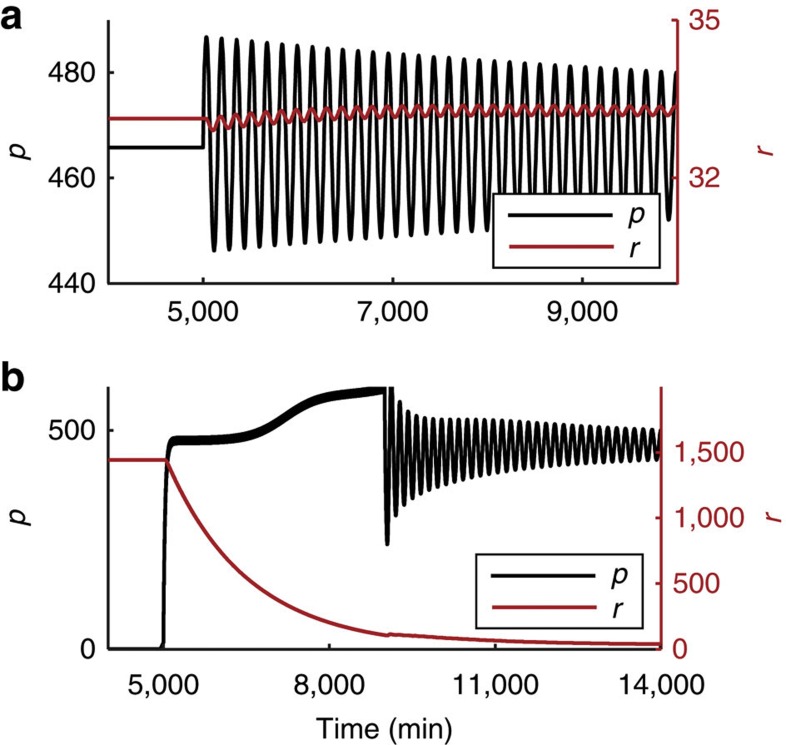
The high Hes1 steady state can be readily excited into transient oscillations. (**a**) The model is initiated in the high Hes1 state in a bistable regime (parameters as default and *p*_1_=220, initial conditions *m*=14.6766, *p*=465.8128, *r*=33.1285). At 5,000 min, the *Hes1* mRNA levels are instantaneously set to a value 10% higher than at this steady state (that is, *m*=16.1433). The system is excited into transient oscillations. (**b**) The model is initiated in the low Hes1 steady state (initial conditions *m*=28.9, *p*=0.4, *r*=1442.6). To excite the system, at *t*=5,000 min, a perturbation of 15 units of protein (an initial 30-fold increase) is added every minute for 4,000 min. Other parameters are *p*_0_=390, *τ*=29 min, *b*_l_=ln(2)/20 min^−1^, *b*_u_=ln(2)/35 min^−1^, *n*_0_=*n*_1_=*m*_0_=*m*_1_=5, *r*_0_=100, *r*_1_=300, *μ*_*p*_=22 min, *μ*_*r*_=1,000 min.

A Hopf bifurcation that gives rise to stable oscillations resides on the top branch of fixed points, and therefore the high Hes1 steady state is a focus near this bifurcation. A consequence of this is that the steady state on the top branch is ‘excitable’ in that it can produce transient oscillations in response to a brief perturbation to the system. An example of this excitability is shown in Fig. 5a, wherein a small, immediate increase in *m* can cause the onset of transient oscillations. In contrast, the steady state on the lower branch does not produce transient oscillations in response to this perturbation, or other, much larger instantaneous changes in *m* (data not shown). We sought an alternative means by which transient oscillations could be initiated from the low Hes1 steady state, and found that a prolonged, pulse perturbation to *p* could achieve this aim, as demonstrated in Fig. 5b. This difference in stability of the top and bottom branches is consistent with their representation of quiescent progenitors and differentiated neurons, respectively. Our interpretation of the result in Fig. 5 is that quiescent progenitors (high steady-state Hes1) are readily re-activated, consistent with the current thinking that they are ‘poised for activation^29^ whereas the differentiated state is robust to change.

### Timing transitions from oscillatory to stable Hes1 dynamics

**Figure 6:**
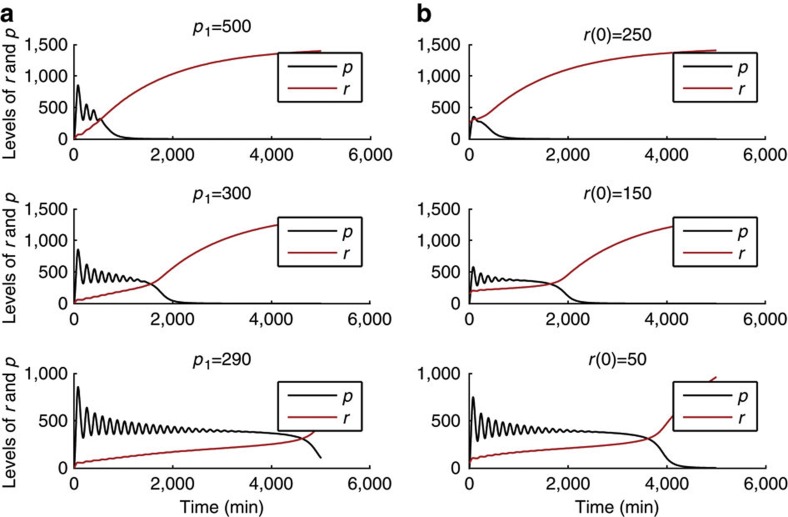
Different durations of oscillations can be found for changing *p*_1_ or the initial levels of *r*. (**a**) Shows simulations with fixed *r*(0)=0 but changing *p*_1_. (**b**) Shows simulations with fixed *p*_1_=290 but changing *r*(0). Here, *m*(0)=0 and *p*(0)=0. Other parameters are *p*_0_=390, *τ*=29 min, *b*_l_=ln(2)/20 min^−1^, *b*_u_=ln(2)/35 min^−1^, *n*_0_=*n*_1_=*m*_0_=*m*_1_=5, *r*_0_=100, *r*_1_=300, *μ*_*p*_=22 min, *μ*_*r*_=1,000 min.

Having shown that high or low steady-state levels of Hes1 can be attained, and even exist in a bistable regime, it is important to consider how the length of time a cell spends as a progenitor before activating differentiation pathways is controlled. We define the length of time that a cell remains as an actively proliferating progenitor as the length of time that Hes1 shows oscillatory dynamics. Introducing miR-9 as a dynamic variable in the system allows miR-9 to increase over time when the production term exceeds the degradation term, recapitulating the increase that is observed experimentally under proliferating conditions. Figure 6 demonstrates two possible ways by which different durations of oscillations can be achieved, before reaching a stable state with low levels of Hes1.

The first of these is a change of parameters. Figure 6a shows that increasing the strength of repression of miR-9 by Hes1 (decreasing *p*_1_) leads to longer transient oscillations. The reason for the lengthening of the transient is that the build up of miR-9 is initially slower, such that the threshold of miR-9 at which oscillations no longer exist is reached at a later time point.

A second way by which the timing of exit from oscillations can be controlled is by the initial amount of miR-9 present in the system, as demonstrated in Fig. 6b. When *r* is initially high, Hes1 is more rapidly repressed to the non-oscillatory state. This is because *r* resides closer to, or even beyond the threshold at which it can be substantially repressed by Hes1 protein and therefore levels of *r* more rapidly increase, further suppressing Hes1.

The transient oscillations of Fig. 6 dampen in amplitude over time. It is therefore important to consider whether the corresponding oscillations in cultured cells would be detectable by our experimental methods. In order to investigate this point, we measured Hes1 expression using bioluminescence imaging of c17.2 cells stably transfected with a Hes1::ub-luciferase reporter. We extracted peaks in an objective way and quantified their amplitude and period. Supplementary Fig. 4 demonstrates that the amplitude and period of the model oscillations are within the range of experimentally quantified values. In the case of dampening oscillations in the example of Supplementary Fig. 4, only the last 3 cycles out of 10 are close to the lower bound of amplitudes that were detected in c17.2 cells, and therefore the majority of these model oscillations would, in principle, be detected experimentally.

**Figure 7:**
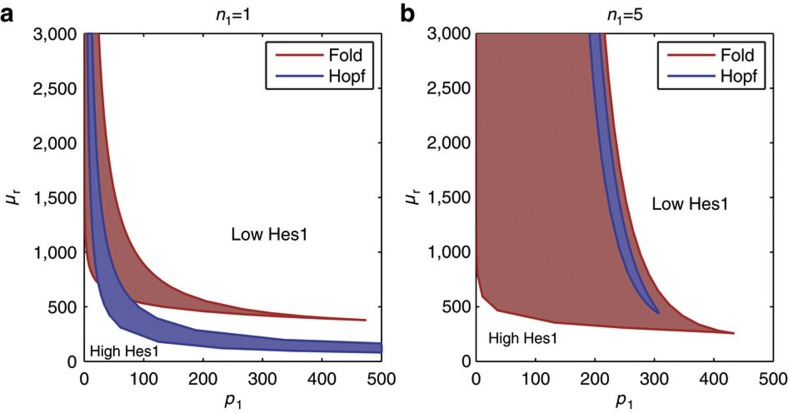
The model can display bistability, sustained oscillations, stable high or low levels of Hes1 depending upon its parameterization. Local bifurcations are shown for changes in the miR-9 degradation rate, *μ*_*r*_, the strength of repression of miR-9 production by Hes1, *p*_1_ and the shape of repression of miR-9 production by Hes1, *n*_1_. The presence of Hopf and fold bifurcations are indicated by blue and red solid lines, respectively. Fold bifurcations lead to the creation or elimination of a pair of fixed points. Blue regions, therefore, indicate the presence of oscillations, while red regions indicate bistability. White regions represent the case of a single steady state with either high or low Hes1 levels (as indicated by the annotation). (**a**) *n*_1_=1. (**b**) *n*_1_=5. Other parameters are *p*_0_=390, *τ*=29 min, *b*_l_=ln(2)/20 min^−1^, *b*_u_=ln(2)/35 min^−1^, *n*_0_=*m*_0_=*m*_1_=5, *r*_0_=100, *r*_1_=300, *μ*_*p*_=22 min.

A comprehensive analysis of the presence of Hopf and fold bifurcations in the full system and its resulting dynamics is shown in Fig. 7 and Supplementary Fig. 5. Hopf and fold bifurcations exist for a range of *n*_1_, *p*_1_ and *μ*_*r*_ values, although their relative position in parameter space is dependent on the choice of *n*_1_.

## Discussion

In this paper, we used mathematical modelling to demonstrate that a negative feedback circuit between miRNA and an autorepressive transcription factor can unify the basic principles of neurogenesis; progenitor maintenance, acquisition of a differentiated state or quiescence and the timing of differentiation. Our model incorporates experimentally constrained parameters, explains a number of experimental observations and provides a unifying theory for cell fate progression, which is likely to be applicable to any organ system that develops from dividing progenitor cells.

Our principal finding is that the amount of active miR-9 available in the cell provides a mechanism to tune the degradation rate of *Hes1* mRNA. From then on, we show that the introduction of miR-9 as a dynamic entity in the Hes1 oscillator has a profound effect on the properties of the regulatory network, over and above the Hes1 oscillator (shown comprehensively in Fig. 7).

First, it gives the Hes1 oscillator the ability to attain either high or low stable Hes1 states. In development, this could account for the ability of progenitor cells to progress either towards differentiation or towards a quiescent, slowly dividing state. Bistability in the system is due to the double-negative feedback loop^30^,^31^, which allows the network to settle into a high or low Hes1 stable state solely by adjusting the initial level of miR-9. Such bistability may account for the ambivalent state of progenitors reported in ref. 17. Alternatively, high or low Hes1 states can be attained by changing the parameters of the system, such as various forms of repression strength. Differential inheritance of miR-9 during mitosis can easily account for varying starting levels of miR-9 in daughter cells. Such differential inheritance could be either stochastic^32^ or the result of fate asymmetric divisions, which are prevalent in the development of the nervous system^2^.

Second, introducing miR-9 as a dynamic entity gives the network the ability to measure lapsed time. In turn, if the transition from oscillatory to stable dynamics marks the transition of cell states, it follows that the network has the ability to time the duration of progenitor maintenance, defined as the time that cells spend in the oscillatory state. We demonstrated that in the model, the timing of transient oscillations can be affected by intrinsic factors (the initial concentration of miR-9) or by factors extrinsic to the model (changes of parameters, see Fig. 6).

The measurement of biological time has been one of the most elusive areas of biology. While progress has been made on understanding cyclical time, found in biological clocks such as the circadian clock^33^, linear time progression has been more difficult to understand, perhaps with the exception of the temporal control of somite segmentation^34^. Our model provides a mechanism by which cyclical time can be converted into linear time. Finally, the observation that the duration of oscillations can vary depending on the parameters of the system or the initial conditions means that although the timer is cell autonomous, it can be externally tuned, by extrinsic factors such as extracellular signalling or cell interactions.

This property endows cells with an innate ability to measure time, while having the flexibility to adjust the timer to external stimuli. We propose that this model reconciles the observation that timed differentiation can be cell autonomous^35^, or can be influenced by extrinsic factors, such as signals from the stem cell niche (reviewed in Okano and Temple^36^). In principle, such extrinsic factors could act in a number of ways, modifying one or more of the parameters of the network such as the strength of repressive interactions. A good example of this is the presence of co-factors (such as Id) that may diminish the autorepressive strength of Hes1 (ref. 37).

Double-negative feedback loops of the sort described here are extremely widespread in biology and particularly in developing systems. Such double-negative loops have been reported between transcription factors (see D-V patterning of the neural tube^38^) or even between transcription factors and miRNAs^31^,^39^. Thus, we predict that the network behaviour that we have described here is widely used by developing systems, the immune system and stem cells, where oscillatory gene expression is also observed^40^,^41^,^42^. Indeed, similar network motifs have been described that account for multi-state gene expression and oscillations due to spatial morphogen gradients^43^. Although the network topology is similar, the output of our model is temporal rather than spatial.

There are several questions that remain to be answered; our model serves to highlight and predict areas of high priority. In many cases, our numerical investigation of the model indicated a switch type behaviour in inhibitory interactions based on the assumption of a relatively steep transition from minimum to maximum repression. Biophysical measurements, such as the precise strength and shape of transcriptional and post-transcriptional repressive interactions and their effect on the duration of oscillations will need to be established *in vivo* in order to test the validity of such assumptions. Future experimentation should also establish quantitative measurements of the abundance of *Hes1* and miR-9 in progenitors and neurons, and examine whether there is differential inheritance during cell division that may predict the fate of daughter cells. Simultaneous imaging of dynamic gene expression and cell fate transition, coupled with optogenetic methods for manipulating oscillations (as recently described in ref. 10) will directly test some of the timing ideas put forward in this paper.

Overall, our data highlights miRNA processing and biogenesis as an important area of research^44^, since the output of our model is sensitive to the concentration of active miR-9. Wider adoption of live imaging techniques and quantitative methods for such highly dynamic gene expression will help the community provide answers to some of the emerging questions.

## Methods

### Experimental methods

c17.2 neural progenitor cells (Sigma-Aldrich) were grown in Dulbecco’s Modified Eagle medium with 10% fetal bovine serum (FBS). To generate stable Hes1 reporter cell lines, pcDNA4-Hes1::ubq-luciferase WT 3′UTR was transfected into c17.2 cells using Lipofectamine 2000 (Invitrogen). Forty-eight hours after transfection, cells were plated into 1 mg ml^−1^ Zeocin (Invitrogen) and maintained in antibiotic selection for 2 weeks. Stable transfectants were plated at low density and individual resistant colonies picked to generate single-cell clones. c17.2 Hes1::ubq-luciferase clones were tested for luciferase expression and positive response to transient NICD overexpression on a FLUOstar Omega plate reader (BMG LABTECH). A representative clone was used for subsequent imaging.

In bioluminescent imaging experiments, c17.2 Hes1::ubq-luciferase reporter cells were plated on 35 mm glass-based dishes (Greiner-Bio One) in sparse (14,000 cells per ml) or confluent conditions (50,000 cells per ml). Sparse cells were allowed to adhere before FBS withdrawal for 3 h and subsequent imaging in the presence of 10% FBS. Confluent cells were serum starved for 24 h before imaging in 10% FBS. 1 mM D-luciferin (Promega) was added prior to imaging and plates were placed on an inverted microscope stage and maintained at 37 °C in 5% CO2. Luminescent images were obtained using a 10 × 0.3 NA air objective and collected with a cooled charge-coupled device camera (Orca II ER, Hamamatsu Photonics). A 30-min exposure and 4 × 4 binning were used.

Bioluminescent movies were analysed using Imaris (Bitplane). Images were subject to smoothing using a 3 × 3 median filter. Individual cells and background regions were tracked manually using the ‘spots’ function. Single-cell bioluminescence values over time were extracted and subsequently analysed for the presence of peaks using MATLAB 7.12 (The MathWorks, Natick, MA, US) (see below).

### Data analysis

We sought to define real peaks and troughs in the data that were distinct from background fluctuations (Supplementary Fig. 7a,b) and spurious changes in ongoing trends (Supplementary Fig. 7b,c).

The first step of the analysis was to calculate background measurements from the data. To this end four control regions of a plate, for each experiment, were identified by the absence of luminescent cells. Sequential changes in luminescence in control regions gave a distribution of values, and we required that real peaks had amplitudes greater than the majority of these. Thus, we set the value of the fluctuation at the 99th percentile in the distribution as a threshold above which the amplitude of real peaks should lie. This process is demonstrated in Supplementary Fig. 6.

All turning point maxima in single-cell data were identified using the MATLAB function ‘findpeaks’, and it was assessed whether their amplitude was greater than the background threshold defined above. We defined amplitude as the change in luminescence measured from the peak to subsequent and previous time points (that is, we considered the descent on either side of the peak), greater than one-time step away. We defined a window length of 15 data points (7.5 h) either side of the peak, which allowed us to include peaks riding on slow changes in luminescence. Shorter windows neglected peaks with long period, whereas larger windows could generate spurious peaks due to chance drops in luminescence at distant time points. Supplementary Fig. 7a,b demonstrate the amplitude thresholding step.

An undesired consequence of the window approach is that peaks occurring as shoulders on larger peaks could still pass the amplitude threshold criterion. Therefore, we required that a significant drop in luminescence (using the same threshold as previously) occurred on either side of the peak before the next peak was encountered. An example of this final processing step is shown in Supplementary Fig. 7c. Troughs were identified by running the algorithm on the negative of the time series.

Having defined ‘real’ peaks and troughs in the data as above, we could proceed to calculate the amplitude and period of oscillations in bioluminescent data. The amplitude was defined as the percentage change in luminescence above background. The mean value over time of each four control regions was calculated, and the lowest of these values designated as a baseline. For each peak, its previous and subsequent troughs were identified and the baseline subtracted from each peak and trough. The two troughs either side of each peak gave two possible estimates for the peak amplitude. The normalized amplitude was defined as the minimum (over these two possible choices) ratio of baseline subtracted peak to trough ((peak–baseline)/(trough–baseline)). The normalized amplitude of the model peaks was calculated in the same way. In this case, the background was taken to be the eventual steady state of protein levels in the model. The period of oscillations in the model and in the data was calculated as the difference in time between each consecutive pair of peaks. All analyses and simulations were performed using MATLAB. For simulations of time series, we used the MATLAB function ‘dde23’ with options ‘RelTol’=10^−5^ and ‘AbsTol’=10^−8^. For history vectors, we used a single value repeated over prior time points. Numerical bifurcation analysis was performed using DDE-BIFTOOL^45^.

## Author contributions

M.G. developed the computational model with input from N.P. M.G. performed the analysis. N.E.P. and T.G. contributed to the development and analysis of the model. C.M. performed the live cell imaging. N.P. supervised the work and co-wrote the manuscript with M.G.

## Additional information

**How to cite this article**: Goodfellow, M. *et al*. microRNA input into a neural ultradian oscillator controls emergence and timing of alternative cell states. *Nat. Commun.* 5:3399 doi: 10.1038/ncomms4399 (2014).

## Supplementary Material

Supplementary Information Supplementary Figures 1-7, Supplementary Note 1 and Supplementary Reference

## Figures and Tables

**Table 1:** Parameters of the model and their default values.

**Parameter**	**Default value**	**Interpretation**	**Reference**
*τ*	29 min	Time delay in Hes1 protein production.	21
*p*_0_	390	Amount of protein required to reduce *Hes1* mRNA transcription by half.	
*n*_0_	5	Quantifies the step-like nature of *G*.	12
*r*_0_	100	Amount of miR-9 required to reduce *Hes1* mRNA degradation rate by half.	
*m*_0_	5	Quantifies the step-like nature of *S*.	
*b*_l_	ln(2)/20 min^−1^	Lower bound for *Hes1* mRNA half-life.	9
*b*_u_	ln(2)/35 min^−1^	Upper bound for *Hes1* mRNA half-life	9
*r*_1_	300	Amount of miR-9 required to reduce Hes1 protein translation rate by half.	
*m*_1_	5	Quantifies the step-like nature of *F*.	
*p*_1_	Varies	Amount of protein required to reduce miR-9 production rate by half.	
*n*_1_	5	Quantifies the step-like nature of *G*_*r*_.	
*μ*_*r*_	1,000 min	Lumped half-life for free miR-9.	
*μ*_*p*_	22 min	Half-life of Hes1 protein.	11
