# Supplementary material for: microRNA input into a neural ultradian oscillator controls emergence and timing of alternative cell states
Source: Nat Commun. 2014 Mar 4;5:3399. doi: 10.1038/ncomms4399 (PMC3959193; doi:10.1038/ncomms4399)
Supplement: Supplementary Information — Supplementary Figures 1-7, Supplementary Note 1 and Supplementary Reference [file ncomms4399-s1.pdf]

Supplementary Information : microRNA input into a neural ultradian oscillator controls emergence and timing of alternative cell states

Marc Goodfellow, Nicholas E. Phillips, Cerys Manning, Tobias Galla, Nancy Papalopulu

Supplementary Figures

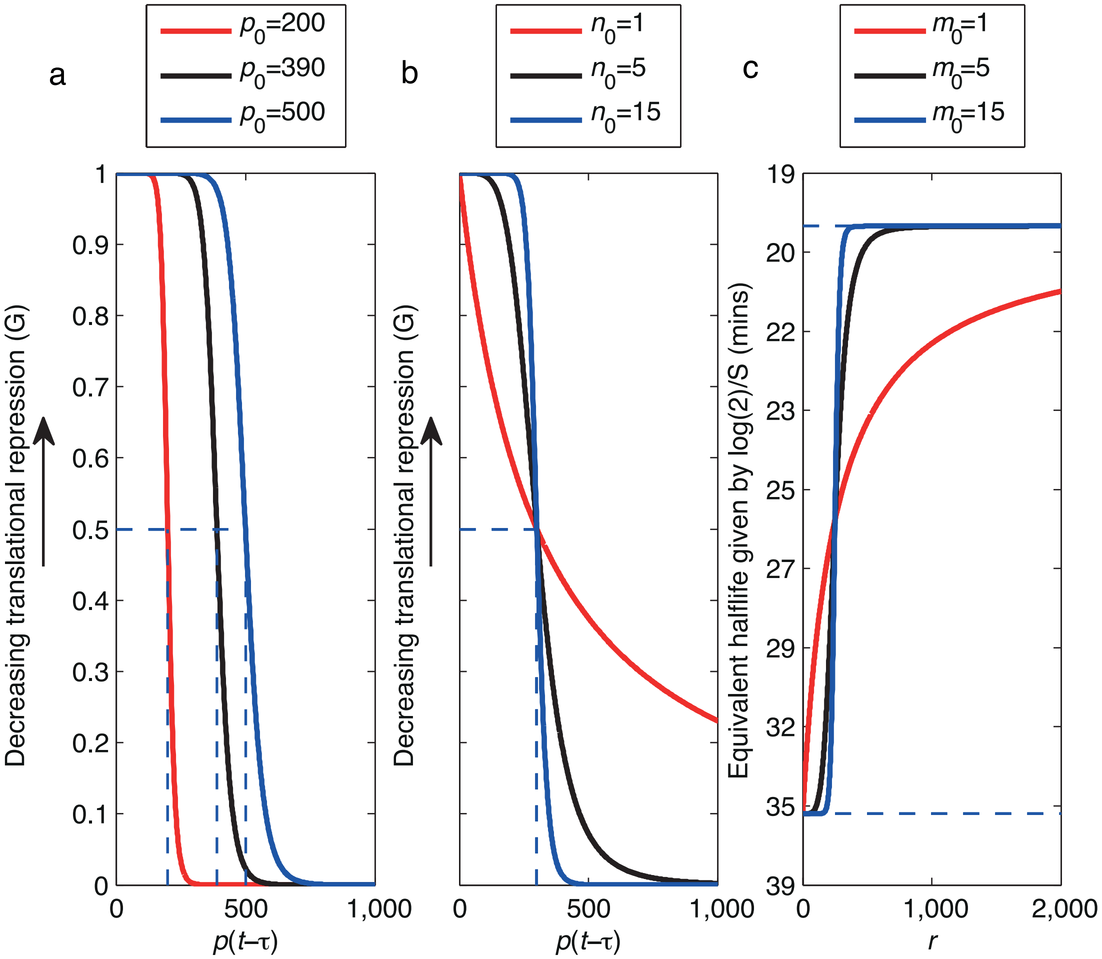

Supplementary Figure 1: Examples of the effect of parameter variations on the functions G and S. a) increasing p_0 leads to a greater value of $p(t-\tau)$ being required to reduce translation of mRNA by half. n_0=5. b) increasing n_0 leads to a more step-like form, i.e. a narrow window over which increases in $p(t-\tau)$ can significantly reduce transcription of *Hes1*. p_0=300. c) the function $S(r)$ is bounded between experimentally determined half-lives (35 and 20 minutes). The effect of changes in m_0 on the form of the relationship between r and S is shown. r_0=250.

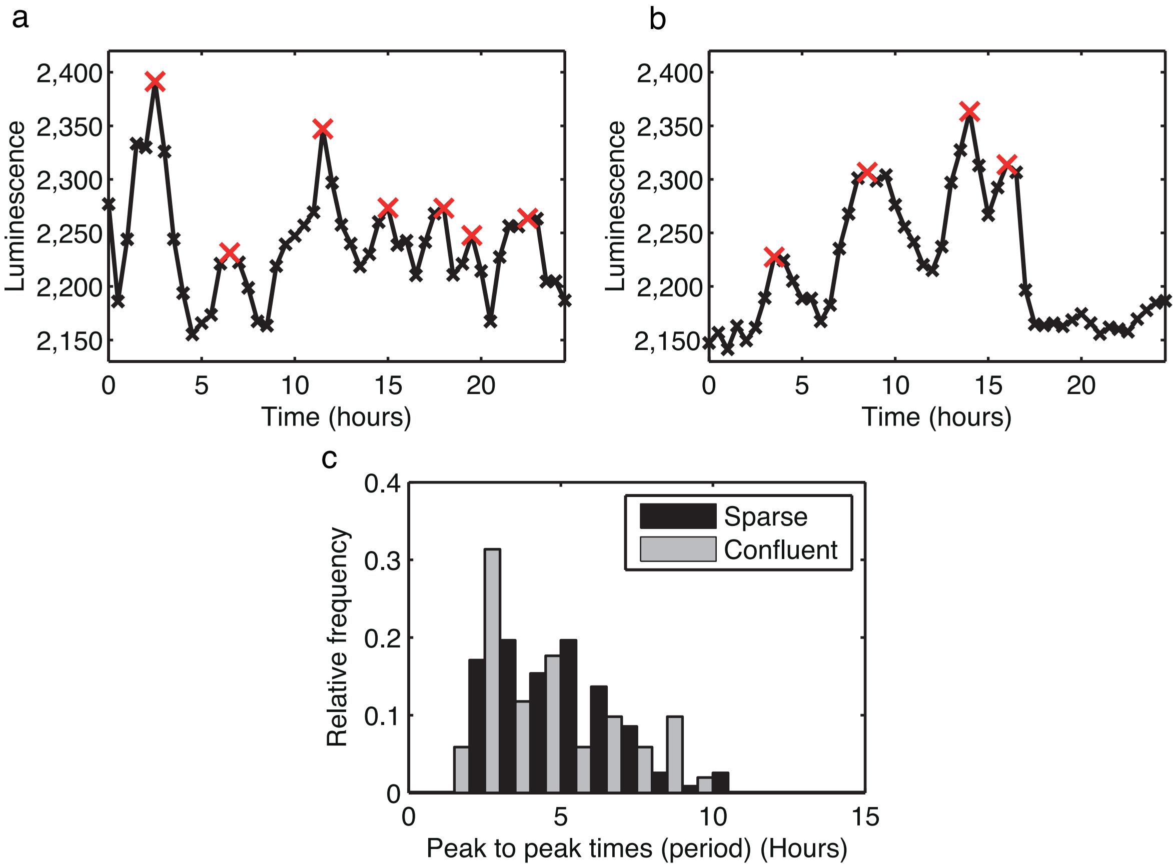

Supplementary Figure 2: Oscillations are found in confluent and sparsely plated cells. c17.2 Hes1::ub-luciferase reporter cells were plated in sparse (a) (14 000 cells/mL, black bars) or confluent (b) (50 000 cells/mL, grey bars) conditions. a) and b) show time series of luminescence for a cell in sparse and confluent conditions, respectively. Original data is shown in black. Extracted peaks are indicated by red crosses. c) histograms of the peak to peak timing under sparse (N=27) and confluent (N=14) conditions.

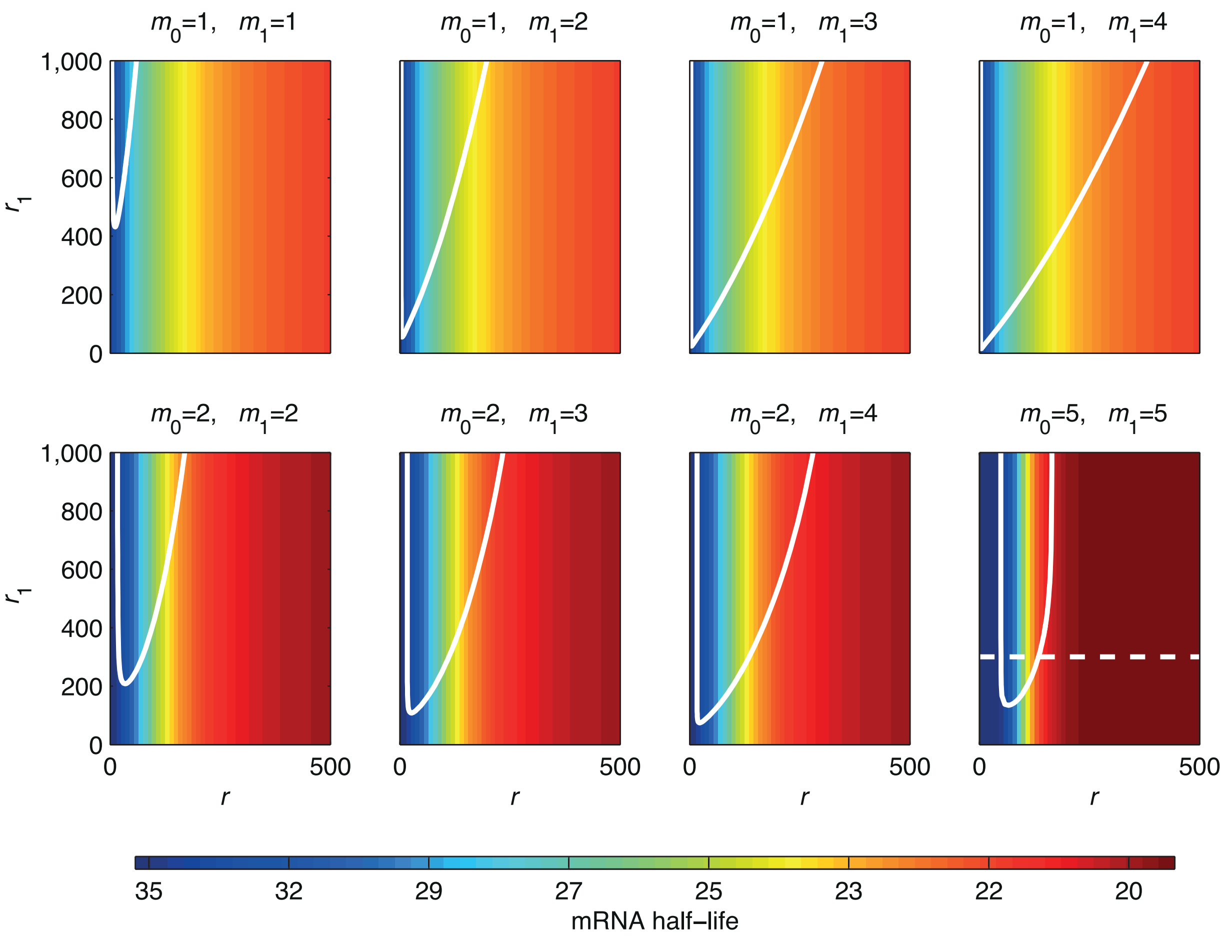

Supplementary Figure 3: Dynamics of the two-variable model including translation repression and *Hes1* mRNA degradation by miR-9. The level of miR-9 (r) is treated as a control parameter, as is the strength of translational repression, r_1. Different combinations of m_0 and m_1 are indicated in the subfigure titles. Other parameters are b_l = ln(2)/20 minutes^{-1}, b_u = ln(2)/35 minutes^{-1}, r_0 = 100, n_0 = 5, μ_p= 22 minutes, p_0 = 390, τ = 29 minutes. Regions of oscillatory solutions exist above the white lines, which represent Hopf bifurcations. The blue-red colour scale indicates the steady state *Hes1* mRNA half-life, which is given by ln(2)/$S(r)$ and the value of which is indicated in the colour bar at the base of the figure. The dashed white line in the bottom right figure demonstrates a window of oscillations corresponding to the experimentally observed half-lives at extreme levels of miR-9 [1].

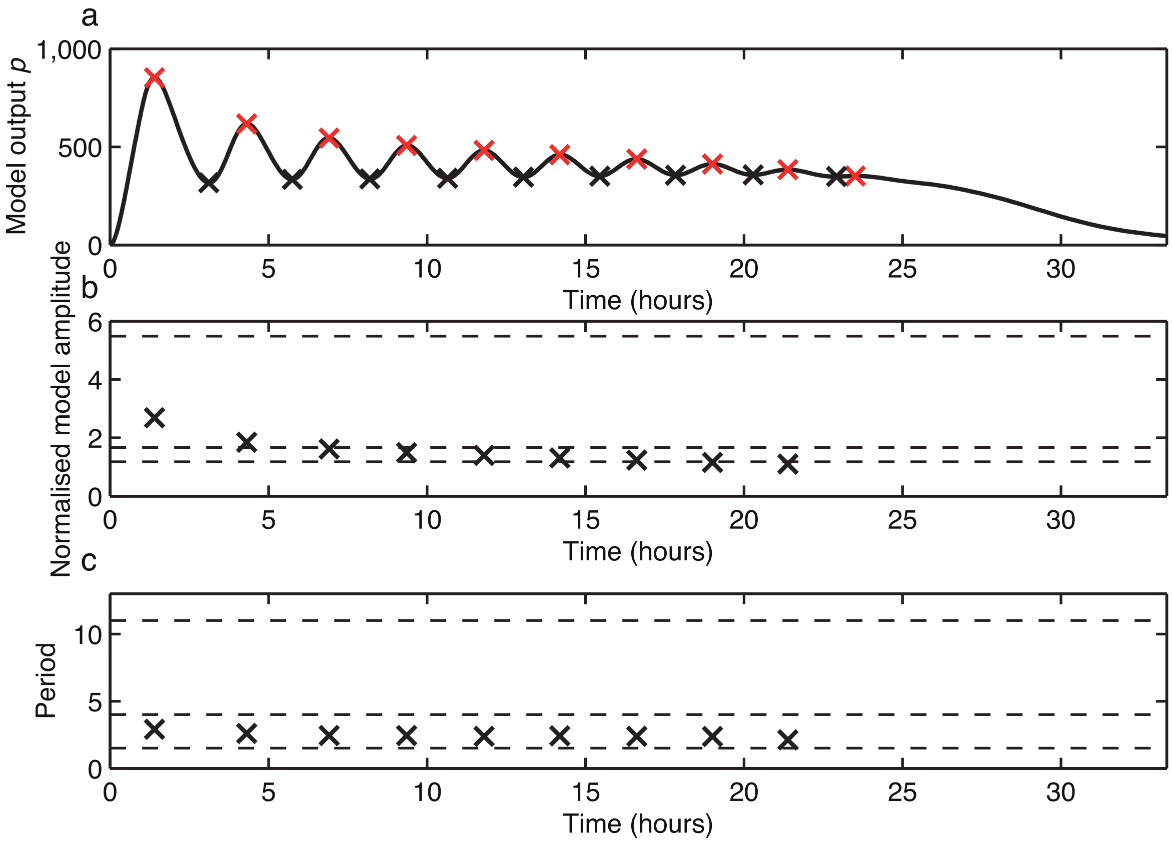

Supplementary Figure 4: Model amplitude and frequency compared to experimental data. a) model dynamics with $p_1 = 330$ and other parameters as default (Table 1). Peaks and troughs are shown by red and black crosses, respectively. b) black crosses represent normalised amplitudes for each cycle of the model output shown in (a). The dashed horizontal lines represent the minimum, median and maximum amplitude of peaks extracted from single cell c17.2 time courses (N=27). c) black crosses represent the period (peak to peak time) for each cycle of the model output shown in (a). The horizontal dashed lines indicate the minimum, median and maximum peak to peak time extracted from single cell c17.2 time courses (N=27).

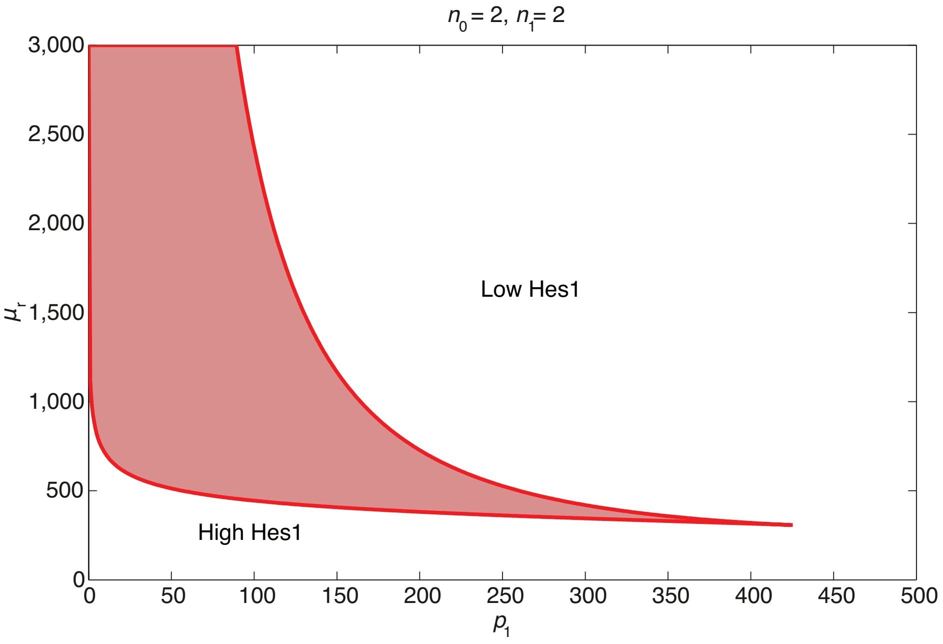

Supplementary Figure 5 : Model bifurcations with n_0=2 and n_1=2 Local bifurcations are shown for changes in the miR-9 degradation rate, μ_r and the strength of repression of miR-9 production by Hes1, p_1 (cf Figure 7 of main text). The presence of fold bifurcations is indicated by red solid lines. The red shaded region indicates the presence of bistability. White regions represent the case of a single steady state with either high or low Hes1 levels. n_0=2, n_1=2. Other parameters are b_l = ln(2)/20 minutes^{-1}, b_u = ln(2)/35 minutes^{-1}, r_0 = 100, r_1 = 300, m_0 = m_1=5, μ_p= 22 minutes, p_0 = 390, τ = 29 minutes. Note that in this case, as expected the Hopf bifurcation is lost, though the region of bistability remains.

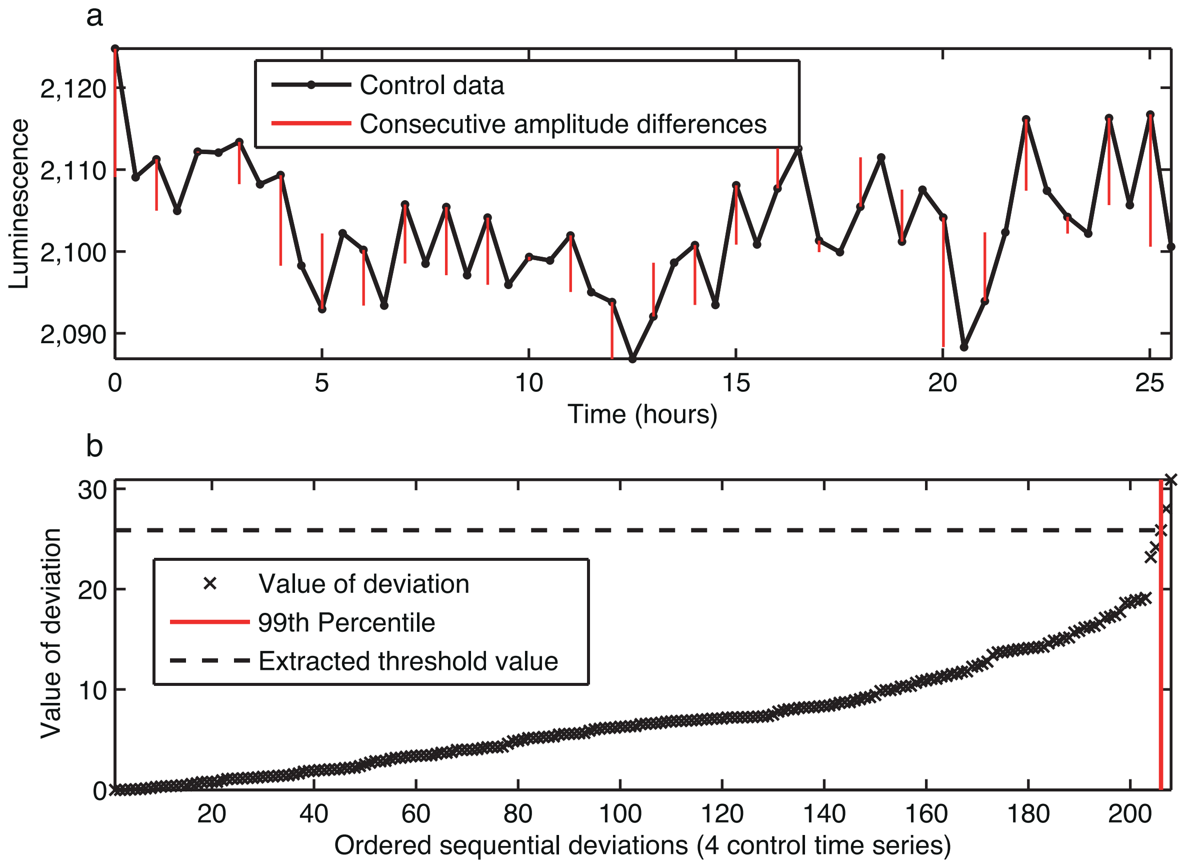

Supplementary Figure 6 : Extraction of amplitude threshold from control data. a) An example of a control time series (i.e. luminescence recorded from a region of the plate with no clearly discernable luminescent cells). Sequential changes in luminescence are shown by red lines (only every second change is indicated for improved visibility). These sequential changes, taken over four control regions for each experiment, form a distribution. An example of such a distribution for an experiment is shown in b). b) The sequential luminescence changes are ordered from lowest to highest and plotted using black crosses. The 99th percentile is indicated by the vertical red line. The value at this point, indicated by the dashed horizontal black line, is taken as the amplitude threshold for this particular experiment. Turning points with an amplitude change above this threshold are identified as peaks.

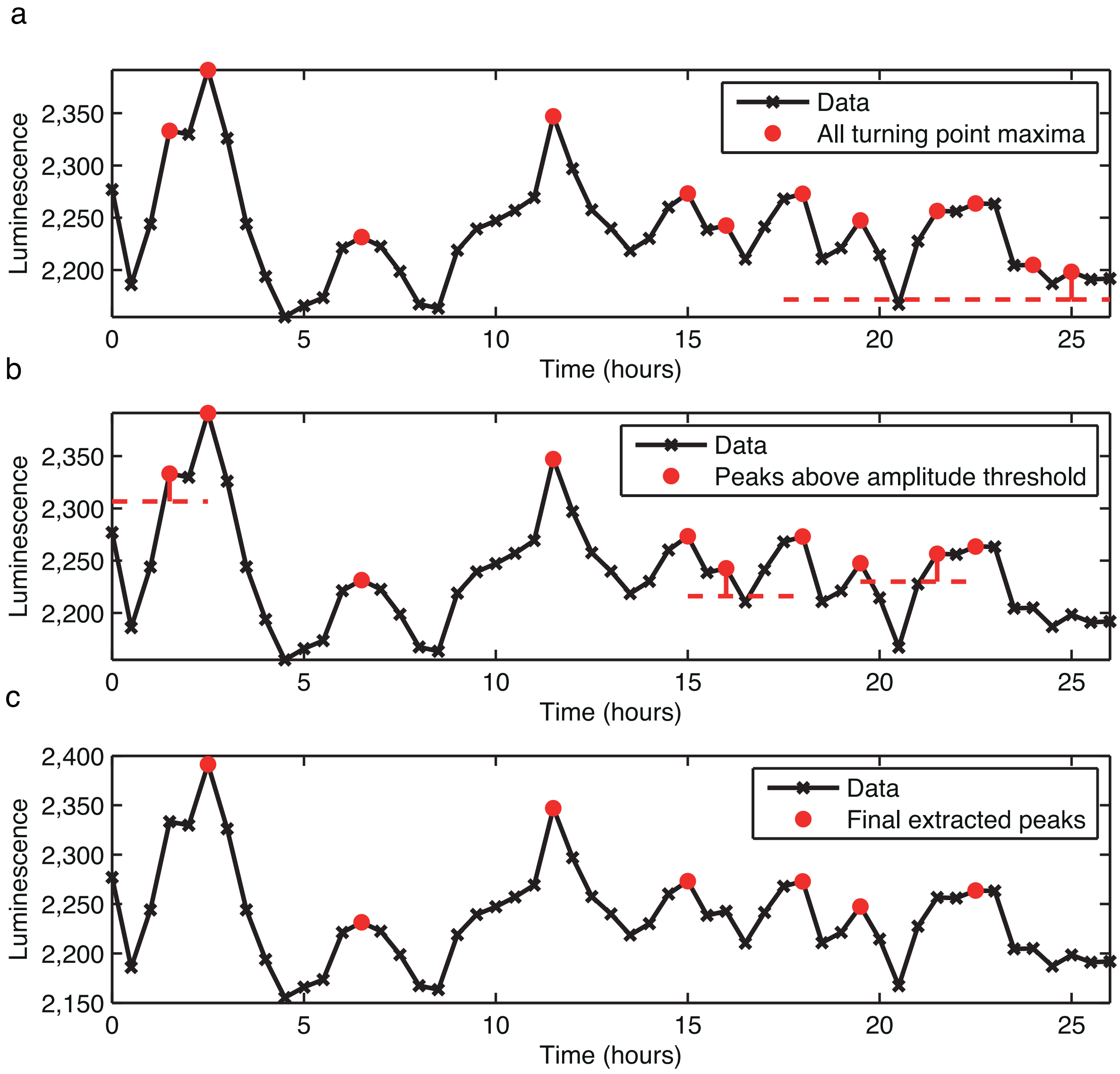

Supplementary Figure 7 : Demonstration of the peak extraction algorithm. a) An example luminescence time series with all turning point maxima denoted by red circles. The dashed red line indicates the amplitude threshold over a 15 data point window for the last peak in the data. Although the data dips below the required luminescence value on the left, it does not on the right and hence this peak is discarded. b) shows peaks that remain after the amplitude thresholding. Example peaks are shown (those with horizontal dashed lines) whose amplitude only reaches threshold after a sequential peak occurs. Thus these peaks are considered 'shoulders' on larger peaks and are removed. c) Resulting peaks after extraction are shown as red circles.

Supplementary Note 1

Here, for convenience, we collate the equations from the Model section of the main text.
The "full model" is governed by the following equations :

$$\frac{dm}{dt} = G(p(t-\tau)) - S(r)m \tag{1}$$

$$\frac{dp}{dt} = F(r)m - \frac{\ln(2)}{\mu_p} p \tag{2}$$

$$\frac{dr}{dt} = G_r(p(t-\tau)) - \frac{\ln(2)}{\mu_r} r \tag{3}$$

where

$$G(p(t-\tau)) = \frac{1}{1+\left(\frac{p(t-\tau)}{p_0}\right)^{n_0}} \tag{4}$$

$$S(r) = b_l + \frac{b_u - b_l}{1+\left(\frac{r}{r_0}\right)^{m_0}} \tag{5}$$

$$F(r) = \frac{1}{1+\left(\frac{r}{r_1}\right)^{m_1}} \tag{6}$$

$$G_r(p(t-\tau)) = \frac{1}{1+\left(\frac{p(t-\tau)}{p_1}\right)^{n_1}} \tag{7}$$

References

[1] Bonev, B., Stanley, P. & Papalopulu, N. MicroRNA-9 Modulates Hes1 Ultradian Oscillations by Forming a Double-Negative Feedback Loop. *Cell reports* 2, 10–18 (2012).
